# FcRn Inhibition in Autoantibody-Mediated Autoimmune Diseases: From Broad Immunosuppression to Precision IgG Modulation

**DOI:** 10.31138/mjr.100826.rhf

**Published:** 2026-09-01

**Authors:** George P. Chrousos, Fotini N. Skopouli, Haralampos M. Moutsopoulos

**Affiliations:** 1 Science Section, National Academy of Athens, Athens, Greece;; 2 Department of Nutrition and Dietetics, Harokopio University, Athens, Greece;; 3 Department of Internal Medicine and Autoimmune Diseases, Euroclinic of Athens, Athens, Greece

**Keywords:** Fc receptors, IgG receptors, immunoglobulin G, autoantibodies, autoimmune diseases, precision medicine

## Abstract

The therapeutic landscape of autoimmune disease is undergoing a conceptual transition. For decades, treatment has relied predominantly on broad immunosuppression, which has substantially reduced morbidity and mortality but remains limited by systemic toxicity and impaired host defence. Advances in molecular immunology have enabled mechanism-based therapies that target pathogenic pathways more selectively while preserving much of normal immune function. Inhibition of the neonatal Fc receptor (FcRn) is a leading example of this approach in IgG-mediated autoimmunity. By interrupting FcRn-dependent IgG recycling, FcRn antagonists accelerate the degradation of circulating IgG, including pathogenic autoanti-bodies, without directly suppressing lymphocyte production or broadly inhibiting cytokine networks. This review summarises the biological basis of FcRn-mediated IgG homeostasis, the rationale for therapeutic FcRn blockade, and its clinical implications across autoantibody-mediated autoimmune diseases. FcRn inhibition exemplifies the transition from empirical immunosuppression to precision immunomodulation and provides a platform for selectively modifying the persistence of disease-causing antibodies.

## KEY MESSAGES

FcRn is a central determinant of IgG persistence. Its pH-dependent recycling pathway protects both physiological IgG and pathogenic IgG autoantibodies from lysosomal degradation.

FcRn antagonism selectively accelerates IgG clearance. Pathogenic autoantibody concentrations fall rapidly without direct lymphocyte depletion or broad suppression of cellular immunity.

Clinical efficacy is mechanism and disease dependent. Success in generalised myasthenia gravis and chronic inflammatory demyelinating polyneuropathy, together with mixed results in other disorders, highlights the need for biomarker-guided patient selection and individualised monitoring.

## WHAT THIS REVIEW ADDS TO PREVIOUS MJR PUBLICATIONS

Previous MJR publications have addressed upstream immune pathways, including type I interferon dysregulation, and the broad unmet needs of systemic autoimmune diseases.^1–3^ This review adds a receptor-centred, cross-disease synthesis of FcRn biology, human pharmacology, pivotal and negative clinical studies, regulatory status, and safety monitoring. It further incorporates the 2025–2026 randomised Sjögren trials of nipocalimab and efgartigimod, linking rapid IgG reduction to clinical response and defining where biomarker-guided FcRn inhibition may complement, rather than simply replace, conventional immunosuppression.

## INTRODUCTION

### A paradigm shift in autoimmune therapeutics

For more than half a century, the management of auto-immune disease has been based on a straightforward therapeutic principle: pathological immune activation should be controlled by suppressing immune function. Glucocorticoids, cytotoxic immunosuppressants, and biological agents directed against cytokines or immune-cell subsets have fundamentally altered the natural history of many autoimmune disorders, transforming previously fatal diseases into chronic, manageable conditions.^4,5^

Despite these achievements, broad immunosuppression remains an imperfect therapeutic compromise. Because conventional agents attenuate both pathological and protective immune responses, their use can increase susceptibility to infection and cause cumulative metabolic, skeletal, cardiovascular, and organ-specific toxicity. The central challenge is therefore not simply to suppress immunity more effectively, but to inhibit the mechanisms that directly drive disease while preserving physiological immune defence.^4,5^

Advances in systems immunology, structural biology, and molecular therapeutics have made this objective increasingly attainable. Autoimmune diseases are now understood as heterogeneous conditions sustained by discrete pathogenic pathways rather than generalised immune activation alone. This shift from broad immunosuppression to precision immunomodulation is among the most important developments in contemporary clinical immunology.^2–5^ FcRn inhibition is a particularly compelling example of mechanism-driven drug development.

### Search strategy

This narrative review followed the MJR-endorsed recommendations for comprehensive biomedical narrative reviews.^1^ MEDLINE/PubMed, Scopus, Web of Science Core Collection, and DOAJ were searched from database inception to 13 August 2026. No publication-year restriction was imposed. English-language full texts were prioritised, while landmark historical reports were retained irrespective of publication date. The core MEDLINE/PubMed strategy combined controlled vocabulary and free-text terms: (“Receptors, Fc”[MeSH] OR “Receptors, IgG”[MeSH] OR “neonatal Fc receptor” OR FcRn) AND (“Autoimmune Diseases”[MeSH] OR “Autoantibodies”[MeSH] OR autoimmun* OR “IgG-mediated”) AND (inhibit* OR block* OR antagonis* OR efgartigimod OR rozanolixizumab OR nipocalimab OR batoclimab). Disease-specific supplementary searches added myasthenia gravis, chronic inflammatory demyelinating polyneuropathy, immune thrombocytopenia, pemphigus, bullous pemphigoid, systemic lupus erythematosus, Sjögren’s syndrome,, and ANCA-associated vasculitis. Equivalent title/abstract/keyword searches were adapted for Scopus and Web of Science; DOAJ was searched with FcRn and neonatal Fc receptor combined with autoimmune disease and each drug name.

Reference lists and forward citations of key mechanistic reviews and clinical trials were examined manually. Regulatory websites and ClinicalTrials.gov were consulted only to verify approvals and current development status. After removal of duplicates, records were screened for direct relevance to FcRn biology, IgG homeostasis, therapeutic FcRn blockade, efficacy, or safety. Priority was given to original mechanistic studies, randomised human trials, systematic reviews, and authoritative contemporary reviews; conference reports and company releases were used only when no peer-reviewed or regulatory source was available. Because this was a narrative rather than systematic review, no formal risk-of-bias assessment or meta-analysis was performed.

## FcRn: FROM NEONATAL TRANSPORT TO ADULT IGG HOMEOSTASIS

The biological concept underlying FcRn was anticipated in 1966, when F. W. R. Brambell proposed that a single receptor could both protect immunoglobulin from degradation and mediate maternal antibody transfer to the newborn.^6^ In 1989, Simister and Mostov isolated the receptor from neonatal rat intestine, demonstrated its structural similarity to major histocompatibility complex class I molecules, and identified it as the neonatal Fc receptor.^7^

FcRn was subsequently shown not to be restricted to the neonatal period. Its expression in adult human intestinal epithelium established that the receptor also performs important functions throughout life.^8^ FcRn is now recognised as an intracellular salvage and transport receptor expressed in endothelial and epithelial cells, placental tissue, and several antigen-presenting cell populations. In addition to IgG, FcRn binds albumin at a distinct site and contributes to the homeostasis of both proteins.^9–16^

FcRn operates through a pH-dependent endosomal recycling pathway. Following nonspecific cellular uptake, IgG enters acidified endosomes, where FcRn binds the Fc region with high affinity. This interaction diverts IgG away from lysosomal degradation and directs the FcRn–IgG complex into recycling or transcytotic compartments. At the cell surface, exposure to physiological pH causes IgG release. This highly efficient salvage pathway contributes to the unusually long serum half-life of IgG, approximately 21 days in humans.^9–16^ **Table 1** summarises the principal steps in this pathway and the consequences of therapeutic blockade.

**Table 1.** FcRn physiology and the consequences of therapeutic blockade.

**FcRn-mediated process**	**Physiological function**	**Consequence of FcRn inhibition**
Cellular uptake	IgG and albumin enter cells mainly through nonspecific endocytosis.	Initial uptake is not prevented; subsequent FcRn-mediated sorting is altered.
Acidic endosomal binding	FcRn binds the IgG Fc region at approximately pH 6.0.	The antagonist prevents or competitively displaces endogenous IgG binding.
Endosomal sorting	FcRn diverts bound IgG away from lysosomes.	Unbound IgG remains in the degradative pathway.
Recycling and release	IgG is returned to the cell surface and released near pH 7.4.	Recycling is reduced and IgG clearance increases.
IgG serum half-life	Maintained at approximately 21 days in humans.	Shortened through accelerated lysosomal catabolism.
Pathogenic autoantibody burden	Pathogenic IgG is protected in the same manner as protective IgG.	Circulating pathogenic IgG concentrations decline.
Albumin homeostasis	FcRn salvages albumin through a site distinct from the IgG-binding site.	Effects are generally limited but may be agent dependent.
Cellular immunity	Not directly governed by FcRn-mediated IgG salvage.	Largely preserved; there is no direct lymphocyte depletion.
Complement-mediated injury	FcRn does not directly activate complement.	May decrease secondarily as complement-fixing autoantibody concentrations fall.
Overall immunological effect	Maintenance of long-term IgG homeostasis and humoral protection.	Selective reduction of IgG with relative preservation of cellular immunity.

FcRn binds IgG and albumin at distinct sites; effects on albumin therefore depend on inhibitor design.

FcRn: neonatal Fc receptor; IgG: immunoglobulin G.^9–20^

## THE BIOLOGICAL PARADOX OF FcRn IN AUTOIMMUNITY

Although FcRn evolved to preserve protective humoral immunity, it cannot distinguish beneficial antibodies from pathogenic autoantibodies. The same recycling pathway that maintains protective IgG therefore also prolongs the persistence of disease-causing IgG.^10–13^

This paradox has major implications for disorders in which autoantibodies are active mediators of tissue injury rather than merely biomarkers. Examples include generalised myasthenia gravis, chronic inflammatory demyelinating polyneuropathy, immune thrombocytopenia, pemphigus, systemic lupus erythematosus, Sjögren’s syndrome, and other IgG-driven diseases. By repeatedly rescuing pathogenic IgG from intracellular degradation, FcRn acts as an unintended molecular stabiliser of autoimmunity.^13,17–35^

Recognition of this mechanism reframed the therapeutic question. Instead of suppressing autoantibody production through broad inhibition of adaptive immunity, it became possible to target the physiological pathway that determines how long circulating pathogenic antibodies persist. ^13,17–20^

## FcRn INHIBITION: SELECTIVE MANIPULATION OF ANTIBODY HOMEOSTASIS

Therapeutic FcRn antagonists block the interaction between FcRn and endogenous IgG. IgG that is not rescued by FcRn remains within the degradative pathway and is delivered to lysosomes, causing a rapid reduction in circulating IgG, including pathogenic autoantibodies. This mechanism does not directly inhibit antibody synthesis, deplete lymphocytes, or broadly suppress cytokine signalling.^13,17–20^

The distinction from conventional immunosuppression is clinically important. Traditional agents primarily reduce immune activity by inhibiting immune-cell activation, proliferation, or survival. FcRn inhibition instead modifies antibody pharmacokinetics. T-cell immunity, B-cell numbers, and many innate immune functions are therefore largely preserved, although IgG-dependent effector functions decline as antibody concentrations fall. The therapeutic target is not the immune cell itself, but a homeostatic pathway that regulates IgG persistence (**Table 2**).^13,20,36,37^

**Table 2.** Comparison of conventional immunosuppression and FcRn inhibition in autoimmune disease.

**Feature**	**Conventional immunosuppression**	**FcRn inhibition**
Primary therapeutic target	Immune-cell activation, proliferation, survival, or cytokine signalling	FcRn-mediated IgG salvage and recycling
Mechanism of action	Suppresses immune-cell function and inflammatory pathways	Prevents endosomal rescue of IgG and accelerates lysosomal degradation
Effect on pathogenic autoantibodies	Usually indirect and variable, through effects on antibody-producing cells	Direct, rapid reduction through enhanced clearance of circulating IgG
Effect on total IgG	Variable and agent dependent	Typically reduced by approximately 60–75%; magnitude is dose and agent dependent
T-cell immunity	Frequently affected	No direct suppressive effect; generally preserved
B-cell number and function	May be suppressed or depleted	No direct B-cell depletion; ongoing IgG synthesis is retained
Innate immune function	May be impaired, depending on the agent	Largely preserved, although IgG-dependent effector activity is reduced
Typical onset of effect	Days to months	Often days to weeks
Principal safety considerations	Infection, metabolic and skeletal toxicity, organ toxicity, and agent-specific complications	Mild-to-moderate infections, headache or administration reactions, and transient hypogammaglobulinaemia; agent-specific monitoring remains necessary
Therapeutic concept	Broad immune suppression	Selective modulation of IgG homeostasis

Effects of conventional immunosuppression vary among drug classes. IgG reduction is approximate and depends on antagonist, dose, formulation, and schedule.

FcRn: neonatal Fc receptor; IgG: immunoglobulin G.^13,17–25,36,37^

## CLINICAL TRANSLATION: EFGARTIGIMOD AS PROOF OF PRINCIPLE

Efgartigimod provided the first successful clinical proof of this concept. It is an engineered human IgG1-derived Fc fragment with increased affinity for FcRn across the relevant pH range. By competitively occupying FcRn, efgartigimod limits the recycling of endogenous IgG and accelerates the degradation of both physiological and pathogenic antibodies.^17^

Clinical studies of FcRn antagonists have demonstrated rapid reductions in total IgG, commonly of approximately 60–75%, together with clinically meaningful improvement in several autoantibody-mediated diseases. The magnitude and duration of IgG reduction vary with the agent, dose, formulation, and treatment schedule. Because protective as well as pathogenic IgG is reduced, respiratory and other infections may occur, and IgG concentrations may require clinical monitoring. Nevertheless, FcRn blockade does not produce the broad cellular immunosuppression characteristic of many conventional therapies.^17–25,32,36,37^

The success of efgartigimod established FcRn as a clinically validated therapeutic target and stimulated development of Fc fragments and monoclonal antibodies directed against the receptor. Efgartigimod, rozanolixizumab, nipocalimab, and batoclimab differ in molecular architecture, FcRn affinity, pH dependence, route, dosing schedule, and effects on albumin.^17–20,22,23,30^ These distinctions caution against assuming complete interchangeability within the class.

Pivotal trials support approvals in generalised myasthenia gravis and chronic inflammatory demyelinating polyneuropathy, while efficacy signals in ITP, Sjögren’s syndrome, and selected other disorders remain jurisdiction-specific or investigational.^21–29,38–45^ Mixed or negative outcomes in pemphigus, bullous pemphigoid, and other development programs show that reducing circulating IgG is not sufficient when pathogenic antibodies are not the dominant reversible driver, tissue damage is advanced, or trial design and background therapy obscure the treatment effect. **Table 3** summarises selected indications and their status as of 13 August 2026.

**Table 3.** Selected IgG autoantibody-mediated diseases and the clinical-development status of FcRn inhibition.

**Disease**	**Principal pathogenic IgG autoantibodies**	**Major mechanism of tissue injury**	**Status of FcRn inhibition (August 2026)**
Generalised myasthenia gravis	Anti-AChR; anti-MuSK; anti-LRP4 in defined subgroups	Complement-mediated junctional injury and receptor loss in AChR disease; disruption of MuSK signalling in MuSK disease	Efgartigimod, rozanolixizumab, and nipocalimab have regulatory approvals in defined antibody-positive populations and jurisdictions.^21–23,39,40^
Chronic inflammatory demyelinating polyneuropathy	Heterogeneous; anti-NF155, anti-CNTN1, anti-CASPR1, or pan-neurofascin antibodies in defined subgroups	Nodal or paranodal dysfunction with immune-mediated peripheral nerve injury	Subcutaneous efgartigimod-based therapy is approved for adults in the United States and other jurisdictions.^24,38^
Primary immune thrombocytopenia	Anti-GPIIb/IIIa and anti-GPIb/IX IgG	Fc receptor-mediated platelet clearance with impaired platelet production	Phase 3 evidence is positive; approval is established in Japan but not uniformly across major jurisdictions.^25^
Pemphigus vulgaris	Anti-desmoglein 3, with or without anti-desmoglein 1 IgG	Disruption of keratinocyte adhesion and acantholysis	No approval. Open-label phase 2 findings were encouraging, but the phase 3 ADDRESS study did not meet its primary endpoint.^26,27,41^
Bullous pemphigoid	Anti-BP180, with or without anti-BP230 IgG	Complement- and inflammatory-cell-mediated dermal–epidermal separation	No approval. A phase 2/3 program has been completed; confirmatory peer-reviewed efficacy evidence is not established.^2^
Systemic lupus erythematosus	Multiple autoantibodies, including anti-dsDNA and anti-Sm IgG	Immune-complex deposition, complement activation, and tissue inflammation	No approval. Phase 2 evidence has been reported and further trials are ongoing; confirmatory peer-reviewed phase 3 evidence is not yet available.^34,43^
Sjögren’s syndrome	Anti-Ro/SSA and anti-La/SSB IgG, among other autoantibodies	B-cell hyperactivity, autoantibody-associated systemic injury, and lymphocytic glandular inflammation	No approval. Randomised phase 2 trials of nipocalimab and efgartigimod reported positive efficacy signals; phase 3 programs are ongoing.^28,29,44–45^
ANCA-associated vasculitis	Anti-PR3 or anti-MPO IgG	ANCA-mediated neutrophil activation and small-vessel inflammation	Mechanistically attractive but clinically unestablished; no approval or confirmed efficacy.^34^

Status is formulation- and jurisdiction-specific and was verified on 13 August 2026; it should be reconfirmed immediately before submission.

AChR: acetylcholine receptor; ANCA: antineutrophil cytoplasmic antibody; BP: bullous pemphigoid; CASPR1: contactin-associated protein 1; CNTN1: contactin 1; dsDNA: double-stranded DNA; FcRn: neonatal Fc receptor; GPIb/IX and GPIIb/IIIa: platelet glycoprotein complexes; LRP4: low-density lipoprotein receptor-related protein 4; MPO: myeloperoxidase; MuSK: muscle-specific kinase; NF155: neurofascin 155; PR3: proteinase 3; Sm: Smith antigen.

## FUTURE PERSPECTIVES

FcRn antagonists illustrate a broader transformation in autoimmune therapeutics: treatment is increasingly selected according to a demonstrable disease mechanism rather than clinical phenotype alone. The most suitable candidates are likely to be disorders in which circulating IgG has a direct and quantitatively important pathogenic role. Biomarkers that identify autoantibody-driven disease, predict response, and guide retreatment intervals will be essential for extending this strategy responsibly.^13,32–35^

The value of FcRn inhibition outside established neurological indications remains under active investigation. Results have differed across diseases and development programs. The positive randomised Sjögren studies provide unusually direct clinical support for a pathogenic contribution of circulating IgG in a systemic rheumatic disease, but they require confirmation in phase 3 trials.^28,29,44,45^ IgG reduction may be insufficient when injury is sustained by long-lived plasma cells, cellular immunity, complement-independent mechanisms, or irreversible structural damage.^26,27,30,31,34^

Long-term safety also requires careful characterisation. Although FcRn inhibition is immunologically selective, repeated treatment lowers protective IgG as well as pathogenic autoantibodies. Clinical practice should integrate infection surveillance, vaccination planning, baseline and on-treatment IgG measurements when appropriate, and individualised decisions about treatment intervals. Available data suggest that patients can mount new vaccine-specific IgG responses during efgartigimod treatment, although pre-existing protective titres fall transiently with total IgG.^36,37^ The reversible pharmacodynamic effect of current agents may permit treatment cycles to be tailored to disease activity and antibody reaccumulation.

## CONCLUSIONS

The development of FcRn inhibitors demonstrates how a fundamental discovery in immunoglobulin biology can redefine clinical practice decades later. By exploiting the physiological mechanism that regulates IgG persistence, FcRn antagonists reduce pathogenic autoantibodies while preserving much of cellular and innate immune function.^13,17–25^

More broadly, FcRn inhibition represents the maturation of precision immunology. The transition from broad immune suppression to mechanism-based modulation is not simply the introduction of another drug class; it is a change in therapeutic philosophy. Its ultimate impact will depend on careful patient selection, long-term safety surveillance, and accurate definition of the diseases in which pathogenic IgG is a central and reversible driver of tissue injury.
